# Identification and Clinical Characterization of Adult Patients with Multigenerational Diabetes Mellitus

**DOI:** 10.1371/journal.pone.0135855

**Published:** 2015-08-19

**Authors:** Ornella Ludovico, Massimo Carella, Luigi Bisceglia, Giorgio Basile, Sandra Mastroianno, Antonio Palena, Salvatore De Cosmo, Massimiliano Copetti, Sabrina Prudente, Vincenzo Trischitta

**Affiliations:** 1 Endocrine Clinical Unit, IRCCS Casa Sollievo della Sofferenza, San Giovanni Rotondo, Italy; 2 Medical Genetics Unit, IRCCS Casa Sollievo della Sofferenza, San Giovanni Rotondo, Italy; 3 Mendel Laboratory, IRCCS Casa Sollievo della Sofferenza, San Giovanni Rotondo, Italy; 4 Department of Experimental Medicine, “Sapienza” University of Rome, Rome, Italy; 5 Unit of Cardiology, IRCCS Casa Sollievo della Sofferenza, San Giovanni Rotondo, Italy; 6 Unit of Medicine, IRCCS Casa Sollievo della Sofferenza, San Giovanni Rotondo, Italy; 7 Unit of Biostatistics, IRCCS Casa Sollievo della Sofferenza, San Giovanni Rotondo, Italy; 8 Research Unit of Diabetes and Endocrine Diseases, IRCCS Casa Sollievo della Sofferenza, San Giovanni Rotondo, Italy; Virgen Macarena University Hospital, School of Medicine, University of Seville, SPAIN

## Abstract

**Background:**

Some patients diagnosed as having type 2 diabetes mellitus (T2DM) are, instead, affected by multigenerational diabetes whose clinical characteristics are mostly undefined.

**Objective:**

1. To identify among patients who had been previously defined as affected by T2DM those, in fact, affected by multigenerational diabetes; 2. After excluding patients carrying the most common MODY genes and mitochondrial mutations, we compared clinical features of remaining patients with those of patients with T2DM.

**Methods:**

Among 2,583 consecutive adult patients who had been defined as affected by T2DM, we looked for those with diabetes in ≥3 consecutive generations. All probands were screened for mutations in six MODY genes (HNF4A, GCK, HNF1A, PDX1, HNF1B and NeuroD1) and for the A3243G mitochondrial mutation. After excluding patients with mutations in one of such genes, we compared clinical features of the remaining 67 patients (2.6% of the whole initial sample) affected by multigenerational “familial diabetes of the adulthood” (FDA) and of their diabetic relatives (n = 63) to those with T2DM (n = 1,028) by generalized hierarchical linear models followed by pairwise comparisons.

**Results:**

Age, age at diagnosis, proportion of hypertension (all p<0.001), and waist circumference (p<0.05) were lower in FDA than T2DM. Nonetheless, the two groups had similar age-adjusted incidence rate of all-cause mortality.

**Conclusions:**

Beside younger age at diagnosis, FDA patients show lower waist circumference and reduced proportion of hypertension as compared to those with T2DM; despite such reduced potential cardiovascular risk factors, FDA patients did not show a reduced mortality risk than patients with T2DM.

## Introduction

Diabetes mellitus imposes high rates of morbidity and mortality worldwide [1]. Thus, better treatment strategies are needed to be set up in order to reduce such tremendous burden affecting health care systems, diabetic patients and their families. One possible strategy for reaching this goal is to deeper understand the different pathogenic backgrounds underlying the several forms of diabetes of the adulthood, including among others maturity onset diabetes of the young (MODY), mitochondrial diabetes, and type 2 diabetes mellitus (T2DM) as well as to unravel new ones, which are still unknown [2]. Differences across these two well-defined forms of diabetes mellitus have been extensively and recently described [3]. Daily clinical experience teaches us that some adult patients who are likely to be diagnosed as having T2DM are, instead, components of families with a multigenerational form of diabetes which still lacks a precise and shared definition [4]. While some of them are certainly affected by MODY or by mitochondrial diabetes which have been misdiagnosed, many others are not. Though some clinical description of such patients have been reported [4], quite surprisingly, a comparison between clinical features with those of patients with T2DM has never been carried out, thus making difficult their identification in the daily clinical set and, eventually, set up novel diagnosis and treatment strategies. In order to try fill this gap of knowledge, the aim of our present work was to firstly identify patients affected by familial diabetes who were not due to the most common (i.e. not private) MODY or mitochondrial mutations, and then to compare their clinical features to those affected by the traditional form of T2DM.

## Methods

### Recruitment of patients and clinical characterization

From a total of 2,583 consecutive Italian diabetic patients who, at our Institution, had been previously defined at a routine clinical visit as affected by T2DM (according to ADA 2003 criteria), we looked for those belonging to families with diabetes in ≥3 consecutive generations. One diabetic patient belonging to each family, and–when possible–their diabetic relatives, were recruited.

One-thousand-twenty-eight unrelated patients with T2DM (as defined according to ADA 2003 criteria), who have been previously recruited and characterized at the same Institution for studying risk factors of all-cause mortality [5], were also included in this study. None of our T2DM patients belonged to multigenerational pedigrees (i.e. diabetes in more than two consecutive generations).

All study patients were interviewed regarding age at diabetes diagnosis, and ongoing antidiabetic, hypolipidemic and antihypertensive treatments. Duration of diabetes was calculated from the calendar year of data collection minus the calendar year of diabetes diagnosis. All these subjects underwent physical examination, including measurements of height, weight (in order to calculate body mass index; BMI), and blood pressure (i.e., two measurements rounded to the nearest 2 mmHg in the sitting position after at least 5 min rest, using an appropriate-sized cuff). Fasting venous blood was sampled from an antecubital vein from all patients for the measurement of fasting glucose, total and HDL cholesterol, triglycerides (i.e. by commercial kits), and HbA1c (i.e. by HPLC Diamat Analyzer; Bio-Rad, Richmond, CA) levels. Hypertension was diagnosed if individuals were currently receiving anti-hypertensive drugs or if their systolic or diastolic blood pressure was ≥130/85 mmHg. Obesity was defined as BMI was ≥30 Kg/m^2^. Micro- and macro-albuminuria was diagnosed if morning urinary albumin creatinine ratio (ACR; mg/mmol) was >2.5/3.5 in males and female and >30 in both sexes, respectively. Dyslipidemia was diagnosed if individuals were currently receiving anti-lipid agents or if they had total cholesterol ≥200 mg/dl, or triglycerides ≥150 mg/dl, or HDL-cholesterol <40 mg/dl for male and <50 mg/dl for female.

### Ethics Statement

The study protocols and the informed consent procedures were approved by the Institutional Ethic Committee of Istituto di Ricovero e Cura a Carattere Scientifico (IRCCS) “Casa Sollievo della Sofferenza”. All participants gave written informed consent.

### Molecular scanning

All probands from families (n = 77) with familial forms of diabetes were screened for mutations in six known MODY genes (i.e. those accounting for the more prevalent forms of MODY) [6]. In addition, also the presence of the A3243G mitochondrial mutation, responsible of the maternally inherited diabetes and deafness (MIDD) was assessed.

Total DNA was extracted from peripheral blood according to standard procedures.

All exons, flanking introns, and minimal promoter regions of HNF4A, GCK, HNF1A, PDX1, HNF1B, and NeuroD1 were amplified from a genomic DNA sample of each proband by polymerase chain reaction (PCR), using gene-specific oligonucleotide primers (S1 Table). Amplicons were subjected to direct sequencing on an automated ABI 3100 (Applied Biosystems, CA), using the ABI Prism BigDye Terminator v1.1 Cycle Sequencing Kit (Applied Biosystems, CA). Results were analysed with Sequencher software v5.0 (GeneCodes, MI).

Mitochondrial DNA fraction was enriched from total DNA sample of each subject by using the REPLI-g Mitochondrial DNA Kit (Qiagen) following manufacturer’s protocol.

The presence of the A3243G mitochondrial mutation was assessed by PCR and direct sequencing using mitochondrial genome-specific oligonucleotide primers as previously reported [7].

### Statistics

Patients’ baseline characteristics were reported as mean ± standard deviation (SD) and percentages for continuous and categorical variables, respectively.

Group comparisons were performed using generalized hierarchical linear models followed by pairwise comparisons. An unstructured covariance matrix was used to account for familiarity.

As for the prospective study for all-cause mortality, the overall survival was defined as the time between enrollment and death. For subjects who did not experience the end point, whose confirmation was obtained by death certificates, survival time was censored at the time of the last available follow-up attempt. Survival analysis was firstly explored estimating and plotting Kaplan-Meier curves which were then compared by log-rank test. To account for the familial design, incidence rates for overall mortality were expressed as the number of events per 100 person-years and compared using a hierarchical Poisson model after adjusting for patients' age and taking into account familiarity as described above.

A p-value <0.05 was considered as statistically significant. All analyses were performed using SAS Release 9.3 (SAS Institute, Cary, NC, USA).

## Results

### Identifying familial diabetes not due to the mot common MODY genes or to mitochondrial mutations

According to our selection criteria, seventy-seven out of the 2,583 (3%) diabetic patients turned out to be affected by familial forms of diabetes and were, therefore, further investigated. Nine out of these probands (11.7%) were MODY gene-positive at mutational analysis (HNF1A: 1 proband carrying the c.864delG, p.Pro291Glnfs*51 mutation and 1 proband carrying the c.1859C>T, p.Thr620Ile mutation; HNF4A: 3 probands, from apparently unrelated families, carrying the c.340C>T, p.Arg114Trp mutation; GCK: 3 probands, from apparently unrelated families, carrying the c.175C>T, p.Pro59Ser mutation and 1 proband carrying the c.775G>A, p.Ala259Thr mutation). One additional proband carried a low-frequency PDX1 functional variant (i.e. Pro239Gln), previously reported in unrelated patients with T2DM [8]. The A3243G mitochondrial mutation was observed in none of the study probands. The remaining 67 patients (2.6% of the whole initial sample) and their affected relatives, whose diabetes is not linked to the most common MODY genes and the A3243G mitochondrial mutation, were defined as having familial diabetes of the adulthood (FDA). In 52 out of 67 of such families, diabetes was inherited from only one side (i.e. mother or father). In addition, 0, 1 and ≥2 relatives from 36, 13 and 18 families were willing to volunteer. Of these, 34 were siblings, 9 were offspring, 9 were parents and 11 were other relatives of the family proband. Information on the number of affected generations and patients as well as the range of age at diabetes diagnosis in each FDA family is reported in S2 Table.

### Comparing clinical features of MODY, FDA and T2DM

**Fig 1:**
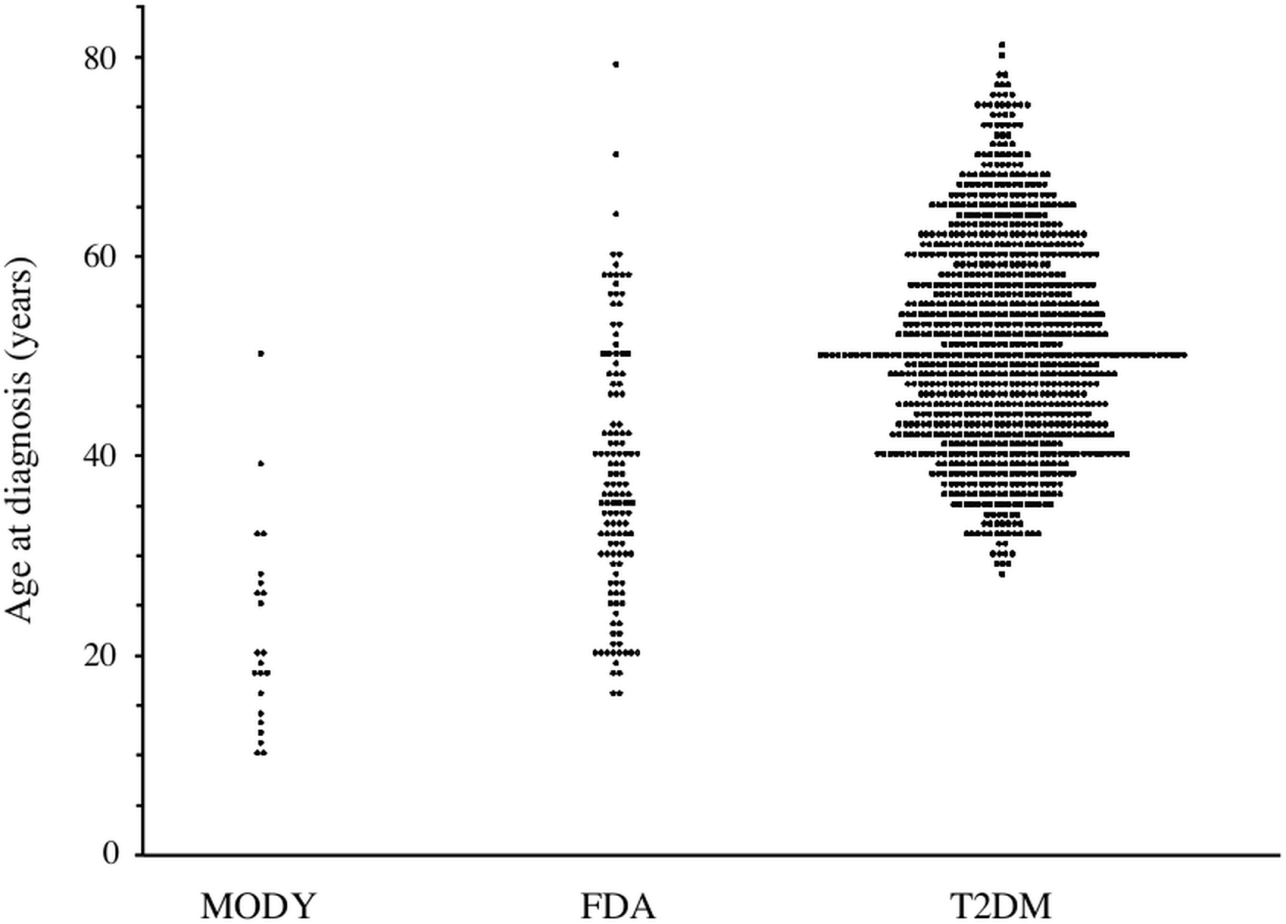
Scatterplot of age at diagnosis in adult patients with MODY, FDA and T2DM.

Individual age at diagnosis in the three study groups are shown in Fig 1. In FDA patients, mean age at diagnosis was thirteen year younger than that of patients with T2DM (Table 1), with only 5 FDA patients (3.85%) as compared to 247 T2DM patients (24.0%), being diagnosed after the age of 60 years (p<0.0001).

**Table 1:** Clinical characteristics of patients (probands and their relatives) with either MODY, FDA or T2DM.

	MODY (N = 22)	FDA (N = 130)	T2DM (N = 1,028)
**Males (%)**	36.4	53.1	50.0
**Age (yrs)**	40.4±15.3	53.3±13.7* ^†^	62.1±9.7
**Age at diagnosis (yrs)**	22.0±10.1	37.7±12.5* ^†^	51.2±10.5
**BMI (Kg/m2)**	24.9±3.4	30.1±6.3^†^	31.1±5.8
**Waist circumference (cm)**	87.7±12.2	97.5±13.6** ^††^	102.4±13.4
**HbA1c (%) (mmol/mol)**	7.1±2.0 (54.0±21.9)	8.7±2.0 (72.0±21.9)^††^	8.7±2.0 (72.0±21.9)
**Anti-hyperglycemic treatment**			
***Diet only (%)***	45.5	12.3	15.8
***OADs (%)***	31.8	42.3	42.5
***Insulin ± OADs (%)***	22.7	45.4^††^	41.7
**Obesity (%)**	13.6	44.4^†^	53.1
**Hypertension (%)**	13.6	48.8* ^†^	84.7
**Dyslipidemia (%)**	63.6	79.8	86.7
**Micro-macro-albuminuria (%)**	9.5	29.6	30.7

Continuous variables were reported as mean + SD, whereas categorical variables are reported as percentages. FDA: Familial Diabetes of the Adulthood; MODY: Maturity Onset Diabetes of the Young; T2DM: Type 2 Diabetes Mellitus; HbA1c: Glycated hemoglobin; OADs: Oral Antidiabetes Drugs

*: p<0.001 vs. T2DM patients

^†^: p≤0.01 vs. MODY patients

**: p<0.05 vs. T2DM patients

^††^: p≤0.05 vs. MODY patients.

Because of the well-known differences across the two clinical entities, no comparisons were carried out between MODY and T2DM.

As a likely consequence of the different age at diagnosis, also age at recruitment was younger in FDA vs. T2DM patients (Table 1). In addition, adiposity measures (i.e. BMI and waist circumference) tended to be lower in FDA vs. T2DM patients, with only waist circumference reaching a statistical significance (Table 1). Finally, the proportion of FDA patients with hypertension was significantly lower being almost half of that in patients with T2DM (Table 1). In contrast, sex distribution, HbA1c, anti-hyperglycemic therapy and proportion of obesity, dyslipidemia, and micro- and macro-albuminuria were similar between the two groups (Table 1).

Conversely, as compared to those affected by MODY, FDA patients were older, showed older age at diagnosis and higher BMI, waist circumference and HbA1c levels (Table 1); in addition, they were more frequently affected by obesity and were on more aggressive anti-hyperglycemic treatments (Table 1).

In a subset of 118 FDA and 838 T2DM patients, mortality rate was assessed. Very likely because of the much younger age in FDA, survival curves were different (S1 Fig; p = 0.04); however, the age-adjusted incidence rate of all-cause mortality, taking into account for the familial design, was virtually identical: 1.93 vs. 1.89 event per 100 person-years in FDA and T2DM (p = 0.93), respectively. This was despite differences between FDA and T2DM in waist circumference (p<0.05) and proportion of hypertension (p<0.001) remained significant also after adjusting for age at diagnosis. No data on mortality rate in MODY patients are available, so that no comparison with FDA patients can be made.

## Discussion

Diabetes mellitus imposes a tremendous burden to health care systems, patients and their relatives [1]. One possible strategy for tackling this burden is to gain new insights about the pathogenic background of specific subgroups of diabetes of the adulthood. A pre-requisite for such ambitious goal is to firstly unravel and characterize new subgroups of diabetes which are not yet known simply because misdiagnosed as being part of established forms of diabetes of the adulthoods ranging from MODY, to mitochondrial diabetes to the most common T2DM. As a matter of fact, daily clinical experience reveals that some adult patients who are likely to be diagnosed as having T2DM are, instead, components of families with a multigenerational form of diabetes which still lacks a precise and shared definition [3]. By carefully re-interviewing 2,583 consecutive adult Italian diabetic patients that, at a routine visit had been defined as affected by T2DM, we identified 77 individuals who were, in fact, affected by familial forms of diabetes. After excluding those carrying mutations in known MODY genes (no mitochondrial mutation was, in fact, observed in our study), 67 patients and their diabetic relatives were defined as affected by a multigenerational form of diabetes mellitus we here propose to define as FDA.

So, the first finding of our study is that in a research-based hospital from Central-Southern Italy, routine activity in the diabetes clinic is likely to overlook the presence of familial forms of diabetes by erroneously overestimating the diagnosis of T2DM. Addressing whether such misdiagnosis is generalizable to many contexts with different cultural and environmental backgrounds is of clinical interest and deserves, therefore, future attempts.

A second finding of our study is that clinical features of FDA patients are somehow intermediate between those from adult MODY patients (who, because of their paucity, were here arbitrarily analyzed as a single group, independently of the genetic cause of diabetes) and those of patients with T2DM. This suggests that FDA patients, rather than carrying mutations grossly affecting insulin synthesis and/or secretion (as in the case of MODY patients) are more likely to carry mutations involved in the fine tuning of insulin secretion or grossly affecting inulin sensitivity. Whether, at least partly, these are the same genes, which, when harboring polymorphisms with a modest biological impact, play a role in T2DM is an intriguing possibility that deserves to be addressed.

In details, as far as the comparison between FDA and T2DM is concerned, our study indicates that patients with FDA are characterized by a younger age at diagnosis so that while an approximately one fourth of patients with T2DM were diagnosed after the age of 60 years, this occurred only in less than 5% of FDA patients. Whether such a cut-off value may be of clinical utility in differentiating between the two forms of diabetes needs to be addressed in further independent populations.

In addition, FDA patients showed a lower waist circumference, and a reduced proportion of hypertension; it is of note that, despite these two latter differences, which were still significant after adjusting for the different age observed between the two groups, the rate of age-adjusted all-cause mortality was not reduced in FDA patients, being virtually identical to that of T2DM patients. Whether FDA have other mortality risk factors we were not able to account for, which may overcome the protective effect of lower abdominal adiposity and blood pressure levels, remains to be understood.

Also to be understood is whether or not FDA patients may benefit from some specific forms of therapies, not only for treating hyperglycemia but also for preventing vascular complications and possibly mortality rate which is disproportionately high if referred to the clearly reduced impact of two established risk factors such as abdominal obesity and hypertension.

This study has some limitations. The first one is represented by the self-reported multigenerational familiarity of diabetes; under this circumstance, underestimation of the proportion of familial forms of diabetes is a likely possibility. The second one goes toward to opposite direction (i.e. the risk of overestimating FDA prevalence) and is secondary to the incomplete molecular diagnosis ascertainment. In fact, other MODY genes so far described to carry very uncommon mutations [9] were not investigated, thus, making impossible to exclude that some of our FDA families were, in fact, affected by such very rare forms of diabetes. Since no anti-GAD antibodies were assessed, it is also possible, tough very unlikely because of the multigenerational inheritance, that some of our FDA patients are, in fact, affected by type 1 diabetes. An additional limitation may be the low number of FDA patients here investigated which may well exposed us to the risk of false negative results and which makes desirable further studies on larger samples.

Finally, a further limitation is the lack of information about most diabetic chronic complications among FDA patients, which impedes us to draw any conclusion about the rate of such events in these patients.

In conclusion, we characterized a familial form of diabetes mellitus, neither due to mutations in the most common MODY genes nor to the A3243G mutation of mitochondrial diabetes, which we propose to define as FDA. Although we have recently reported that in one of our families a severe mutation in the *APPL1* gene causes hyperglycemia [10], further studies are needed to unravel and dissect the whole genetic background of FDA. From a clinical point of view, it remains to be understood whether the less pronounced abdominal adiposity, the lower risk of hypertension or, conversely, the earlier diagnosis, which at a given age results in a longer disease duration, shape a different cardiovascular risk in FDA as compared to T2DM patients and whether different treatment strategies have to be preferred across these different diabetes subtypes.

## Supporting Information

S1 Fig Survival curves by Kaplan-Meier estimator for all-cause mortality in patients with FDA or T2DM. (PDF)

S1 Table Gene-specific oligonucleotide primers for all exons, flanking introns, and minimal promoter regions of HNF4A, GCK, HNF1A, PDX1, HNF1B, and NeuroD1. (DOCX)

S2 Table Number of affected generations and patients as well as range of age at diagnosis in each FDA family. (DOCX)
