# Supplementary figures and images for: Identification and Clinical Characterization of Adult Patients with Multigenerational Diabetes Mellitus
Source: PLoS One. 2015 Aug 19;10(8):e0135855. doi: 10.1371/journal.pone.0135855 (PMC4545999; doi:10.1371/journal.pone.0135855)

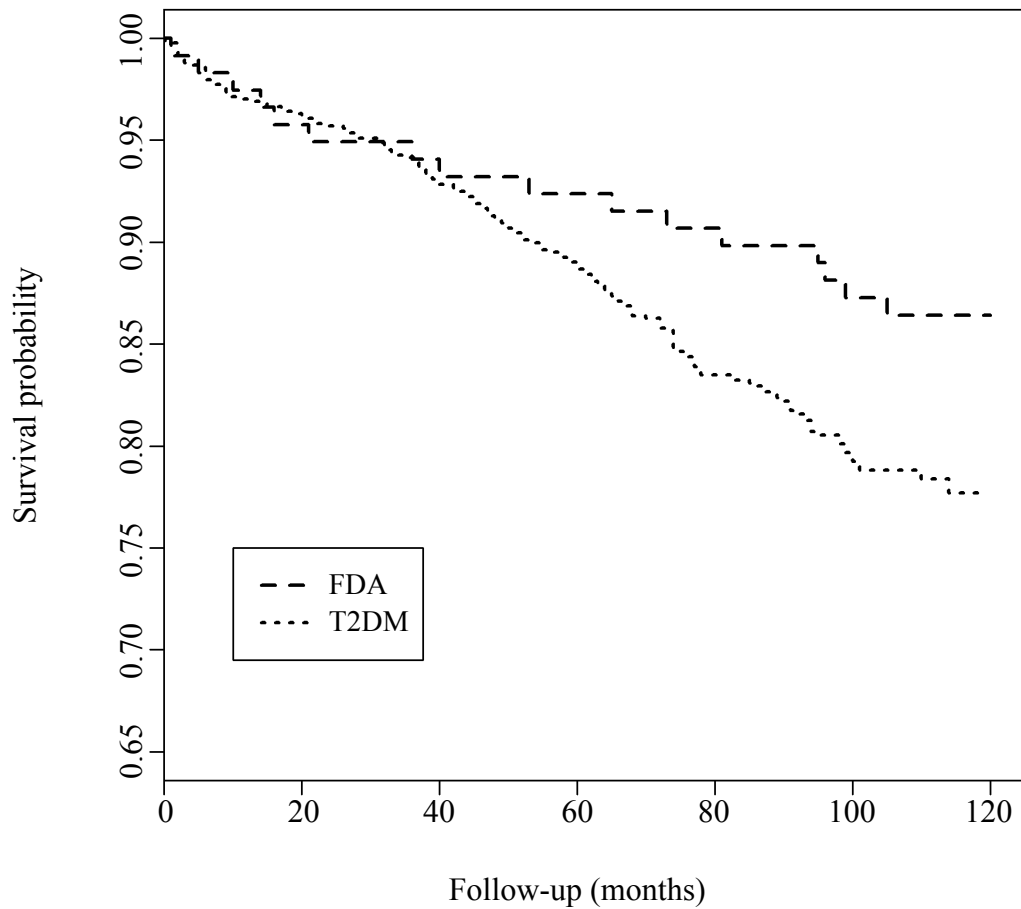

Supplement: S1 Fig — (PDF) [file pone.0135855.s001.pdf]
