# Supplementary material for: Identification and Clinical Characterization of Adult Patients with Multigenerational Diabetes Mellitus
Source: PLoS One. 2015 Aug 19;10(8):e0135855. doi: 10.1371/journal.pone.0135855 (PMC4545999; doi:10.1371/journal.pone.0135855)
Supplement: S1 Table — (DOCX) [file pone.0135855.s002.docx]

**Supporting Information**

**S1 Table. Gene-specific oligonucleotide primers for all exons, flanking introns, and minimal promoter regions of HNF4A, GCK, HNF1A, PDX1, HNF1B, and NeuroD1.**

| Gene | ReFSeq | Primer name | Sequence | TA °C |
| --- | --- | --- | --- | --- |
| HNF4A | NM_000457.3 | HNF4A_1F | 5'-CAGATCTTCCCAAGAGGACGG-3' | 60 |
|  |  | HNF4A_1R | 5'-GTTGAGTGCCAAAGATCTGC-3' |  |
|  |  | HNF4A_2F | 5'-CCGCAAGGCTCCCTTAGATG-3' | 60 |
|  |  | HNF4A_2R | 5'-GAAGAGCCTTGAAGGCCCTG-3' |  |
|  |  | HNF4A_3F | 5'-TAGTTCTGTCCTAAGAGGAG-3' | 60 |
|  |  | HNF4A_3R | 5'-TGCCACTGAGTCATAAAGTG-3' |  |
|  |  | HNF4A_4F | 5'-CCTCATCAGTCACAGACACC-3' | 60 |
|  |  | HNF4A_4R | 5'-CTGCTCCAGGGCCTGGCTGG-3' |  |
|  |  | HNF4A_5F | 5'-GGACAGAGAGTGCGGGAGGG-3' | 60 |
|  |  | HNF4A_5R | 5'-CAAGCCAGTCCACGGCTATA-3' |  |
|  |  | HNF4A_6F | 5'-AGCGTCACTGAGTTGGCTAC-3' | 60 |
|  |  | HNF4A_6R | 5'-CCTTGCATGCCACCATGTGA-3' |  |
|  |  | HNF4A_7F | 5'-CCAGCTATCTTGCCAACTTA-3' | 60 |
|  |  | HNF4A_7R | 5'-TGTGAACTCAGTGTACAGAA-3' |  |
|  |  | HNF4A_8F | 5'-TCTTGACTCCCCAGATGCTC-3' | 60 |
|  |  | HNF4A_8R | 5'-ACTGTGTGAGGCCTGTCT-3' |  |
|  |  | HNF4A_9F | 5'-AGGCCTGAGGTCTGCATCC-3' | 60 |
|  |  | HNF4A_9R | 5'-TGGTTCTACCTTCTAGACTC-3' |  |
|  |  | HNF4A_10F | 5'-AAAGGCTGGAATTTTGAGCA-3' | 60 |
|  |  | HNF4A_10R | 5'-CACCAGGTGCTCTCTTAGGG-3' |  |
| GCK | NM_000162.3 | GCK_1F | 5'-TCAGAAGCCTACTGGGGAAG-3' | 60 |
|  |  | GCK_1R | 5'-CCTTCTCAAAGAGCCTGTGC-3' |  |
|  |  | GCK_2F | 5'-GTGCAGATGCCTGGTGACAG-3' | 62 |
|  |  | GCK_2R | 5'-CACAGCTGCTTCTGGATGAG-3' |  |
|  |  | GCK_3F | 5'-AGCCCAAGGCCAGCCTGTGG-3' | 62 |
|  |  | GCK_3R | 5'-TCAGGACTAGCTGGGCCCTG-3' |  |
|  |  | GCK_4F | 5'-TGAGGAATAGCTTGGCTTGA-3' | 60 |
|  |  | GCK_4R | 5'-TTGAAGGCAGAGTTCCTCTG-3' |  |
|  |  | GCK_5F | 5'-CCTGCCTCCAGTATATGTTAGC-3' | 60 |
|  |  | GCK_6R | 5'-GAGCCTCAGCAGTCTGGAAG-3' |  |
|  |  | GCK_7F | 5'-AGGAACCAGGCCCTACTCCG-3' | 60 |
|  |  | GCK_7R | 5'-CATCTGCCGCTGCACCAGAG-3' |  |
|  |  | GCK_8F | 5'-CCGGCTTCCACCTGCATG-3' | 60 |
|  |  | GCK_8R | 5'-GACCAAGTCTGCAGTGCCCG-3' |  |
|  |  | GCK_9F | 5'-CCTGGAGAACGAGAGGCC-3' | 60 |
|  |  | GCK_9R | 5'-CGAGAAGAGGACTACGAAAT-3' |  |
|  |  | GCK_10F | 5'-GGGCGCCCGGTAATGAATGT-3' | 60 |
|  |  | GCK_10R | 5'-GTCCTGACAAGGCCAGGCCT-3' |  |
| HNF1A | NM_000545.5 | HNF1A_1F | 5'-TGCAAGGAGTTTGGTTTGTG-3' | 60 |
|  |  | HNF1A_1R | 5'-GCCCCTCTAGGCTCTCCTG-3' |  |
|  |  | HNF1A_2F | 5'-CTGAGCAGATCCCGTCCTT-3' | 60 |
|  |  | HNF1A_2R | 5'-CCCTCCCAGGGAAGATGC -3' |  |
|  |  | HNF1A_3F | 5'-TCACGGCTTTCTGTGCCTGC-3' | 58 |
|  |  | HNF1A_3R | 5'-CTTAGGTTCAAGTATTCTCA-3' |  |
|  |  | HNF1A_4F | 5'-CCAGGACAGGGTTCCTCTGA-3' | 62 |
|  |  | HNF1A_4R | 5'-GCTGTCACTGGGACAGTCCT-3' |  |
|  |  | HNF1A_5F | 5'-AAGTGCTGAGGGCTGTGGAG-3' | 60 |
|  |  | HNF1A_5R | 5'-CTAGGGACTGCTCCAGAATCT-3' |  |
|  |  | HNF1A_6F | 5'-GATTCTGGAGCAGTCCCTAG-3' | 60 |
|  |  | HNF1A_6R | 5'-AATGAATGAGTCCCAGTGGCT-3' |  |
|  |  | HNF1A_7F | 5'-CTTGGGAGGTCTTGGGCAGG-3' | 60 |
|  |  | HNF1A_7R | 5'-ACTGCAATGCCTGCCAGGCA-3' |  |
|  |  | HNF1A_8F | 5'-AGTCTTGAGGCCTGGGACTA-3' | 60 |
|  |  | HNF1A_9R | 5'-ACAGTGACGGACAGCAACAG-3' |  |
|  |  | HNF1A_10F | 5'-AGCCTTGTTTGCCTCTGC-3' | 60 |
|  |  | HNF1A_10R | 5'-CCAAGCAGGCAGTACAGG-3' |  |
| PDX1 | NM_000209.3 | PDX1_1.1F | 5'-CCTGGGCCTAGCCTCTTAGT-3' | 60 |
|  |  | PDX1_1.1R | 5'-ACCTCGTACGGGGAGATGT-3' |  |
|  |  | PDX1_1.2F | 5'-AGCTTTACAAGGACCCATGC-3' | 60 |
|  |  | PDX1_1.2R | 5'-TTAGTCCGACCCGGGATAAT-3' |  |
|  |  | PDX1_2.1F | 5'-TTGAAGGGGTTGGGCTGCGT-3' | 60 |
|  |  | PDX1_2.1R | 5'-GCCGCCGCGCTTCTTGTCCT-3' |  |
|  |  | PDX1_2.2F | 5'-GAGCTGGCTGTCATGTTGAA-3' | 60 |
|  |  | PDX1_2.2R | 5'-AGTGGTTGAAGCCCCTCAG-3' |  |
| HNF1B | NM_000458.2 | HNF1B_1F | 5'-TTCTTTTTCCGTCCTTGGAA-3' | 60 |
|  |  | HNF1B_1R | 5'-GACTTCTCTGGTGGGAAACG-3' |  |
|  |  | HNF1B_2F | 5'-CCCTAACCATCTGCTTGTCTG-3' | 60 |
|  |  | HNF1B_2R | 5'-AGAGGGCAAAGGTCACTTCA-3' |  |
|  |  | HNF1B_3F | 5'-GTCTGTCTGCTGAGTGAAGG-3' | 60 |
|  |  | HNF1B_3R | 5'-TAGTGTCTCAATATCCCAGG-3' |  |
|  |  | HNF1B_4F | 5'-AAGACTGCTGTGATTGTGTG-3' | 60 |
|  |  | HNF1B_4R | 5'-AACCAGATAAGATCCGTGGC-3' |  |
|  |  | HNF1B_5F | 5'-GTGCCGAGTCATTGTTCCAG-3' | 60 |
|  |  | HNF1B_5R | 5'-AGGCAGGCCTTGTGAGAAGT-3' |  |
|  |  | HNF1B_6F | 5'-GCTCTTTGTGGTCCAAGTCC-3' | 60 |
|  |  | HNF1B_6R | 5'-TTCTCCCTGCCCCCAAGT-3' |  |
|  |  | HNF1B_7F | 5'-GATGGCATCCATCCACCTCT-3' | 60 |
|  |  | HNF1B_7R | 5'-AAGTTCAGACCCAGAGAGGG-3' |  |
|  |  | HNF1B_8F | 5'-GCCTGTGTATGCACCTTGATT-3' | 60 |
|  |  | HNF1B_8R | 5'-TGCTTGCCACAACCTCTGCA-3' |  |
|  |  | HNF1B_9F | 5'-AACTAATGGCCCATGACCCTGCCA-3' | 60 |
|  |  | HNF1B_9R | 5'-AGGACAGACAGGAGTCCTTG-3' |  |
| NeuroD1 | NM_001030003.2 | NeuroD1_1.1F | 5'-TTCGCAAGCATTTGTACAGG-3' | 60 |
|  |  | NeuroD1_1.1R | 5'-CTTGGGCTTTTGATCGTCAT-3' |  |
|  |  | NeuroD1_1.2F | 5'-AAACCATGAACGCAGAGGAG-3' | 60 |
|  |  | NeuroD1_1.2R | 5'-CCAGGCGACTGGTAGGAGTA-3' |  |
|  |  | NeuroD1_1.3F | 5'-CAATCCTCGGACTTTTCTGC-3' | 60 |
|  |  | NeuroD1_1.3R | 5'-TGCAGCAGTAGTACCCAAAGG-3' |  |
