# Supplementary material for: Identification and Clinical Characterization of Adult Patients with Multigenerational Diabetes Mellitus
Source: PLoS One. 2015 Aug 19;10(8):e0135855. doi: 10.1371/journal.pone.0135855 (PMC4545999; doi:10.1371/journal.pone.0135855)
Supplement: S2 Table — (DOCX) [file pone.0135855.s003.docx]

**Supporting Information**

**S2 Table. Number of affected generations and patients as well as range of age at diagnosis in each FDA family.**

| Family  code number | Affected generations (n) | Affected patients (n) | Age at  diagnosis (range) |
| --- | --- | --- | --- |
| 1 | 4 | 13 | 27-59 |
| 2 | 3 | 5 | 16-60 |
| 3 | 3 | 8 | 25-52 |
| 4 | 3 | 11 | 30-45 |
| 5 | 3 | 8 | 21-50 |
| 6 | 3 | 5 | 25-60 |
| 7 | 3 | 3 | 25-40 |
| 8 | 3 | 4 | 23-50 |
| 9 | 3 | 5 | 27-33 |
| 10 | 3 | 3 | 33-58 |
| 11 | 5 | 14 | 16-65 |
| 12 | 3 | 7 | 35-60 |
| 13 | 3 | 6 | 43-70 |
| 14 | 4 | 6 | 35-85 |
| 15 | 4 | 8 | 42-80 |
| 16 | 3 | 3 | 20-65 |
| 17 | 4 | 8 | 20-37 |
| 18 | 3 | 12 | 22-60 |
| 19 | 4 | 7 | 32-56 |
| 20 | 4 | 5 | 31-60 |
| 21 | 3 | 6 | 25-66 |
| 22 | 3 | 4 | 24-48 |
| 23 | 3 | 5 | 31-56 |
| 24 | 3 | 2 | 16-55 |
| 25 | 3 | 6 | 32-58 |
| 26 | 3 | 7 | 21-33 |
| 27 | 4 | 12 | 32-55 |
| 28 | 3 | 8 | 30-45 |
| 29 | 3 | 8 | 38-56 |
| 30 | 3 | 6 | 34-50 |
| 31 | 4 | 18 | 37-60 |
| 32 | 3 | 4 | 41-60 |
| 33 | 4 | 4 | 28-65 |
| 34 | 3 | 11 | 30-70 |
| 35 | 3 | 6 | 34-60 |
| 36 | 3 | 7 | 37-53 |
| 37 | 3 | 4 | 30-50 |
| 38 | 3 | 6 | 22-63 |
| 39 | 3 | 5 | 30-65 |
| 40 | 3 | 5 | 29-50 |
| 41 | 4 | 7 | 30-42 |
| 42 | 4 | 10 | 28-53 |
| 43 | 4 | 7 | 25-70 |
| 44 | 3 | 8 | 26-42 |
| 45 | 4 | 12 | 28-62 |
| 46 | 3 | 5 | 33-47 |
| 47 | 4 | 11 | 28-60 |
| 48 | 3 | 11 | 22-57 |
| 49 | 3 | 4 | 20-55 |
| 50 | 3 | 11 | 36-90 |
| 51 | 3 | 10 | 20-70 |
| 52 | 3 | 3 | 32-60 |
| 53 | 3 | 7 | 20-64 |
| 54 | 3 | 6 | 38-58 |
| 55 | 3 | 9 | 20-56 |
| 56 | 4 | 7 | 34-40 |
| 57 | 4 | 8 | 35-50 |
| 58 | 4 | 6 | 30-60 |
| 59 | 3 | 6 | 30-79 |
| 60 | 3 | 9 | 20-70 |
| 61 | 5 | 15 | 36-70 |
| 62 | 3 | 8 | 32-70 |
| 63 | 4 | 14 | 37-60 |
| 64 | 3 | 4 | 26-56 |
| 65 | 3 | 5 | 25-60 |
| 66 | 4 | 10 | 18-41 |
| 67 | 4 | 6 | 41-* |

* In family n. 67, age at diagnosis was available only in one patient.
